# Intraindividual dynamics of transcriptome and genome-wide stability of DNA methylation

**DOI:** 10.1038/srep26424

**Published:** 2016-05-19

**Authors:** Ryohei Furukawa, Tsuyoshi Hachiya, Hideki Ohmomo, Yuh Shiwa, Kanako Ono, Sadafumi Suzuki, Mamoru Satoh, Jiro Hitomi, Kenji Sobue, Atsushi Shimizu

**Affiliations:** 1 Division of Biomedical Information Analysis, Iwate Tohoku Medical Megabank Organization, Iwate Medical University, 2-1-1 Nishitokuta, Yahaba-cho, Shiwa-gun, Iwate 028-3694, Japan; 2 Division of Biobank and Data Management, Iwate Tohoku Medical Megabank Organization, Iwate Medical University, 2-1-1 Nishitokuta, Yahaba-cho, Shiwa-gun, Iwate 028-3694, Japan; 3 Department of Physiology, Keio University School of Medicine, 35 Shinanomachi, Shinjuku-ku, Tokyo 160-8582, Japan; 4 Community Medical Supports and Health Record Informatics, Iwate Tohoku Medical Megabank Organization, Iwate Medical University Disaster Reconstruction Center, 2-1-1 Nishitokuta, Yahaba-cho, Shiwa-gun, Iwate 028-3694, Japan; 5 Division of Biomedical Information Analysis, Institute for Biomedical Science, Iwate Medical University, 2-1-1 Nishitokuta, Yahaba-cho, Shiwa-gun, Iwate 028-3694, Japan; 6 Deputy Executive Director, Iwate Tohoku Medical Megabank Organization, Disaster Reconstruction Center, Iwate Medical University, 2-1-1 Nishitokuta, Yahaba-cho, Shiwa-gun, Iwate 028-3694, Japan; 7 Department of Anatomy, School of Medicine, Iwate Medical University, 2-1-1 Nishitokuta, Yahaba-cho, Shiwa-gun, Iwate 028-3694, Japan; 8 Executive Director, Iwate Tohoku Medical Megabank Organization, Disaster Reconstruction Center, Iwate Medical University, 2-1-1 Nishitokuta, Yahaba-cho, Shiwa-gun, Iwate 028-3694, Japan; 9 Department of Neuroscience, Institute for Biomedical Science, Iwate Medical University, 2-1-1 Nishitokuta, Yahaba-cho, Shiwa-gun, Iwate 028-3694, Japan

## Abstract

Cytosine methylation at CpG dinucleotides is an epigenetic mechanism that affects the gene expression profiles responsible for the functional differences in various cells and tissues. Although gene expression patterns are dynamically altered in response to various stimuli, the intraindividual dynamics of DNA methylation in human cells are yet to be fully understood. Here, we investigated the extent to which DNA methylation contributes to the dynamics of gene expression by collecting 24 blood samples from two individuals over a period of 3 months. Transcriptome and methylome association analyses revealed that only ~2% of dynamic changes in gene expression could be explained by the intraindividual variation of DNA methylation levels in peripheral blood mononuclear cells and purified monocytes. These results showed that DNA methylation levels remain stable for at least several months, suggesting that disease-associated DNA methylation markers are useful for estimating the risk of disease manifestation.

Cytosine methylation at CpG dinucleotides is a key epigenetic mechanism that regulates gene expression. The dynamics and stability of DNA methylation are essential for cellular differentiation and the maintenance of cell type-specific gene expression patterns, respectively. Recent research on genome-wide DNA methylation has revealed various types of differentially methylated regions (DMRs), such as tissue-specific DMRs (T-DMRs)^1^,^2^. Notably, DNA methylation within T-DMRs contributes to formation of the transcriptomes that govern cell/tissue specificity by regulating the expression of transcription factors and their downstream target genes^2^. Thus, DNA methylation provides an additional method of gene regulation that leads to functional differences in cells and tissues that characterize individual diversity.

From this viewpoint, large-scale research on human disease-associated epigenetic variations of DNA methylation—referred to as epigenome-wide association studies (EWAS)—have recently attracted much attention^3^. In EWAS, the stability of DNA methylation is important, since DNA methylation profiles are often measured only once for each individual due to issues with cost and sampling. Generally, DNA methylation is considered to be a stable epigenetic marker for specific gene expression patterns that are inherited by daughter cells following mitosis. In fact, previous studies have shown that the relative proportions of genome-wide DNA methylation can be stable for months, years, or decades^4^,^5^.

Alternatively, an increasing number of reports have identified dynamic switching in DNA methylation. For example, DNA methylation is tightly regulated to control *CYP27B1* gene transcription in response to intracellular hormonal changes^6^. In skeletal muscle, acute exercise promotes the expression of exercise-responsive genes in parallel with hypomethylation on their promoters^7^. However, these findings are inconsistent with the prevailing views on DNA methylation as a stable epigenetic marker and suggest the possibility that baseline methylation levels frequently fluctuate in response to various factors, thereby regulating gene expression. Nevertheless, DNA regions have been detected that exhibit a high degree of transient DNA methylation within individuals and can vary over the course of 72 h^8^.

In this study, we evaluated the transience of DNA methylation and its contribution to transcriptome regulation in a genome-wide manner. For this, we collected 24 blood specimens from two individuals over the course of 3 months and obtained methylome and transcriptome data from peripheral blood mononuclear cells (PBMCs) and isolated monocytes. Based on these data, we examined the contribution of DNA methylation to the short-term dynamics of gene expression.

## Results

### Intraindividual gene expression and DNA methylation profiling over 3 months

**Figure 1:**
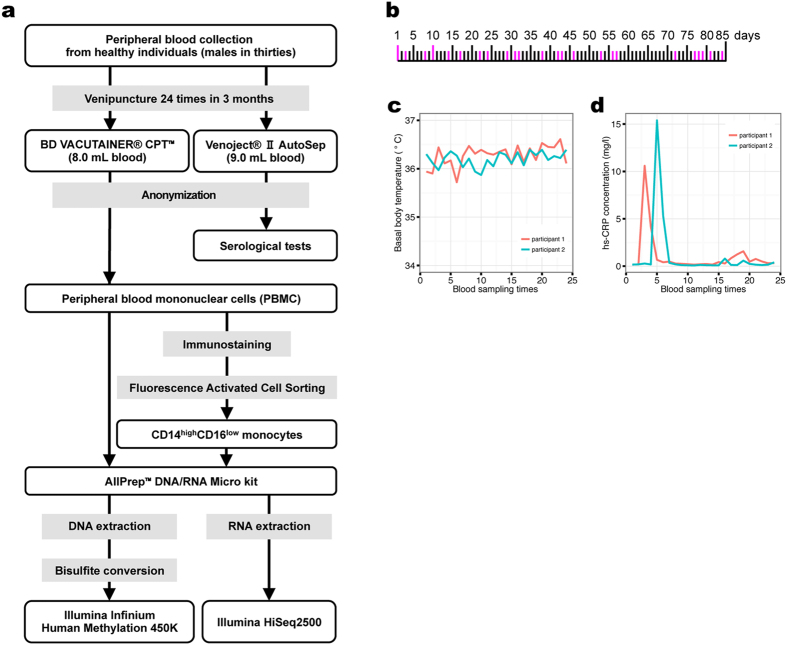
Study design. (**a**) Study workflow. (**b**) Schedule of blood collection. Magenta bars indicate the day of blood collection. (**c**,**d**) Participant’s health monitoring. Body temperatures (**c**) and high-sensitivity C-reactive protein (hs-CRP) serum levels (**d**) of the participants over the blood collection period.

**Figure 2:**
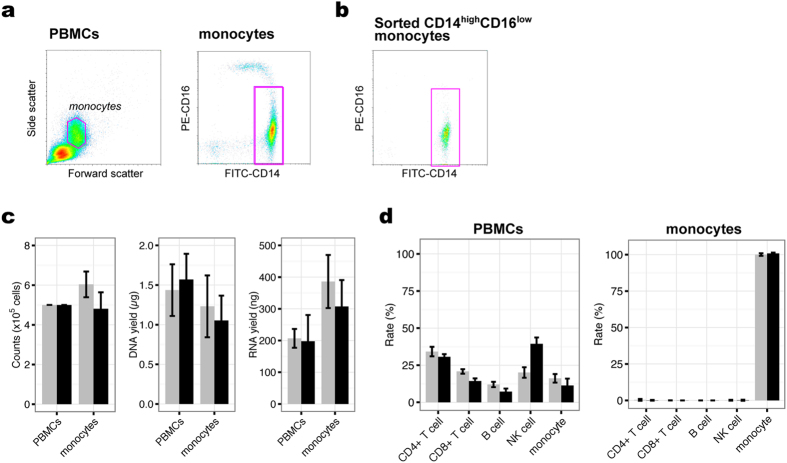
Sample summaries. (**a**) Monocyte gating strategy. Typical light-scatter density-plot of PBMCs (left) and CD14^high^CD16^low^ monocytes were isolated from the monocyte-containing gate based on CD14 and CD16 expression (right). (**b**) Isolated CD14^high^CD16^low^ monocyte population (96.7% ± 1.0% purity). (**c**) Cell numbers (left) and DNA (middle) and RNA (right) yields obtained for each cell population. (**d**) Estimated cellular composition for each sample as determined by reference information on cell-specific DNA methylation signatures using the “estimateCellCounts” function implemented in the minfi package. Bars in (**c**,**d**) represent mean ± standard deviation (grey, participant #1; black, participant #2).

The workflow of this study is illustrated in Fig. 1a. Peripheral blood was collected according to the schedule shown in Fig. 1b. The participants’ health was monitored by basal body temperatures and C-reactive protein serological testing (Fig. 1c,d; Supplementary Table 1). Of note, while the high-sensitivity C-reactive protein (hs-CRP) level was moderately high at the time of the third and the fifth collections in the first and the second participant, respectively, these individuals did not have fever and were thus presumed to be healthy. After harvesting PBMCs, classical CD14^high^CD16^low^ monocytes^9^ were isolated by fluorescence-activated cell sorting, yielding a highly pure population (96.7% ± 1.0% in participant #1 and 96.4% ± 1.0% in participant #2) (Fig. 2a,b). DNA and RNA were then harvested from the respective cell population for epigenetic and transcriptomic analyses, respectively (Fig. 2c).

To examine the intraindividual gene expression variability in PBMCs and monocytes, RNA-Seq analysis was performed for the PBMC and monocyte populations. On average, each transcriptome yielded 25.6 ± 1.7 (mean ± SD) million single-end reads with a mapping ratio of 92.7% ± 3.6%.

We next analyzed the genome-wide DNA methylation profiles of PBMCs and monocytes with a DNA methylation array. Over 483,900 probes (≥99.7%) were detected with detection *p-*values < 0.01 in all samples, indicative of high-quality array data. After quantile normalization, we estimated cell-type composition for each sample by statistical methods using reference information on cell-specific DNA methylation signatures (Fig. 2d).

### Dynamically expressed genes reflect cell function characteristics

**Figure 3:**
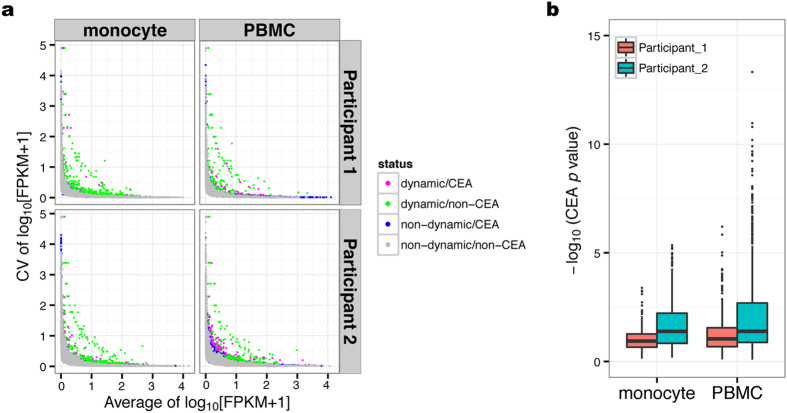
Extraction of dynamically expressed genes. (**a**) Dynamically expressed genes were defined as coefficient of variation (CV) outliers for each gene with 0.05-interval bins of the average of log_10_ [FPKM + 1]. (**b**) Association analysis between cell-type composition and variation in the expression of dynamically expressed genes in each cell population. The vertical axis shows the distribution of the negative log_10_ (*p* values) obtained from linear regression and ANOVA testing.

To identify genes with dynamic changes in expression, the coefficient of variation (CV) for each gene was used as an indicator of changes in expression, and baseline outliers were defined as dynamically expressed genes (Fig. 3a). Of 19,586 genes, 448 (2.3%) and 511 (2.8%) dynamically expressed genes were identified in monocytes of participant #1 and participant #2, respectively. In PBMCs, 329 (1.7%) and 450 (2.3%) genes were identified as dynamically expressed genes in participants #1 and #2, respectively.

Since isolated cell populations generally consist of several cell types, it is unclear whether the observed differences in gene expression are representative of the actual cellular expression level or the change in cell-type composition. Thus, to assess the effect of cell-type composition on gene expression, linear regression and ANOVA testing were used to detect Cell-type composition/Expression Associations (CEAs). Notably, ~20% of the gene expression was associated with cell-type composition in monocytes with a significance level of 0.05 (Table 1a, Fig. 3b). In the PBMC population, the distribution of *p-*values was greater than that observed in monocytes, with 30–35% of gene expression being associated with cell-type composition (*p* < 0.05) (Table 1b, Fig. 3b). In these genes associated with cell-type composition, 2–5% were detected as dynamically expressed genes (Table 1). These results suggested that cell-type composition imparts a great effect on the observed levels of gene expression, particularly in PBMCs, and that cell sorting is effective at reducing this effect.

Next, we investigated whether the dynamic genes unassociated with cell-type composition (dynamic non-CEA genes) were enriched in any specific pathway. As shown in Table 2a, the dynamic non-CEA genes identified in monocytes were enriched in several immune response pathways, reflecting characteristic of monocyte functions (Table 2a). Interestingly, especially in participant #1, the increased expression of some genes corresponded to peaks in serum hs-CRP, indicating that the changes in the expression of the dynamic non-CEA genes reflected actual biological reactions (Supplementary Fig. 1). In PBMCs, dynamic non-CEA genes from participant #1 were enriched only in the cytokine-cytokine receptor interaction pathway (Table 2b).

We also performed the same analysis excluding data for the day that the participants had a high hs-CRP level (Fig. 1d; Day 3 for participant #1 and Day 5 for participant #2). In monocytes, the number of dynamic genes was less than half of the dynamic genes obtained from the full-data in participant #2, whereas those in participant #1 did not change (Table 1, Supplementary Table 2). Moreover, most of the genes showing variation corresponding to the hs-CRP levels could not be detected in the enriched pathway with dynamic non-CEA genes (Supplementary Table 3).

### DNA methylation does not contribute to dynamic changes in gene expression in *cis*

**Figure 4:**
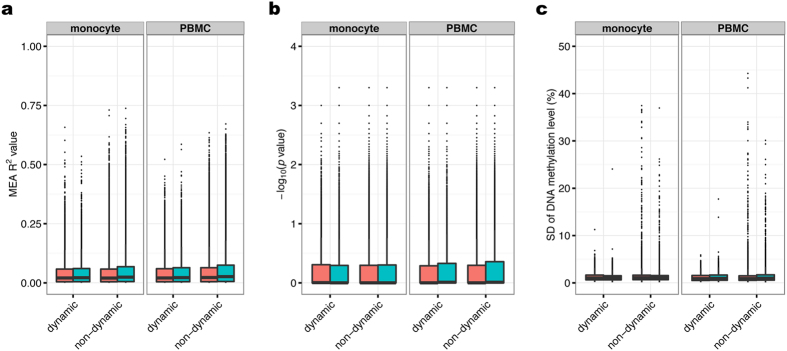
Contribution of DNA methylation to gene expression. (**a**) The distribution of the R^2^ values—representing the proportion of gene expression variation due to DNA methylation—was computed by linear regression analysis. (**b**) The distribution of negative log_10_ (*p* values) obtained from Spearman’s rank correlation and permutation test. (**c**) The standard deviation distribution for DNA methylation at each CpG site near the dynamic non-CEA genes. (**a**,**b**) All FPKM and beta values were converted to relative values using the values of Day 1 as a reference.

To determine to what extent the DNA methylation could regulate dynamic gene expression in *cis*, R^2^ values were obtained from Methylation/Expression Association (MEA) analysis by linear regression. For example, an R^2^ value of 0.1 is indicative of a 10% variance in gene expression that can be explained by the DNA methylation of a CpG site. To exclude any effects of cell-type composition changes, CEA genes were removed from this analysis. Considering the differences in the definitions of FPKM and beta values (the former is relative to the total amount of RNA and the latter is the ratio of methylated probe intensity to total signal intensity), we converted these values to relative values using the values of Day 1 as a reference. In monocytes, the median R^2^ value for both participants was 0.02 (interquartile range [IQR] = 0.00–0.06) for pairs of dynamically expressed genes and neighboring CpG sites (Fig. 4a), demonstrating that only ~2% of the dynamic changes in gene expression could be explained by variations in DNA methylation. Moreover, no significant differences were observed between the distributions of R^2^ values for the pairs of the dynamically and non-dynamically expressed genes and their neighboring CpG sites (participant #1: median = 0.02, IQR = 0.00–0.06, *p* = 0.14; participant #2: median = 0.02, IQR = 0.01–0.07, *p* = 1.00) (Fig. 4a), suggesting that the variation of DNA methylation levels was unlikely to contribute to the dynamics of gene expression in monocytes. The same results were obtained in PBMCs of participant #1 and #2: dynamically expressed genes: median = 0.02 (IQR = 0.00–0.06) in both participants; non-dynamically expressed genes: median = 0.02 (IQR = 0.01–0.06) for participant #2 and 0.03 (IQR = 0.01–0.07) for participant #2, with a *p-*value of 1.00 for the difference between the two R^2^ distributions in both participant. Additionally, no marked differences were identified among CpG site annotations (e.g., promoters, exons, and introns) in either monocytes or PBMCs (Supplementary Fig. 2). We also performed the MEA analysis by linear regression with adjustment of cell-type composition without stratifying the genes by CEAs, and the same results were obtained (Supplementary Fig. 3); suggesting that only ~2% of the dynamic gene expression could be explained by DNA methylation.

Next, in consideration of the non-parametric FPKM and beta values, which did not normally distributed (Supplementary Fig. 4), we performed MEA analysis by applying Spearman’s rank correlation and a permutation test. In monocytes, the median *p* values were 0.99 (IQR = 0.49–1.00) for participant #1 and 1.00 (IQR = 0.51–1.00) for participant #2 for pairs of dynamically expressed genes and neighboring CpG sites (Fig. 4b). There were no significant differences between the *p* value distributions for dynamically and non-dynamically expressed gene/CpG pairs. In PBMCs, the results were similar: the median of dynamically expressed genes was 1.00 in both participants (IQR = 0.51–1.00 in participant #1 and 0.48–1.00 in participant #2). In monocytes, 320 (participant #1) and 208 (participant #2) CpG sites were associated with dynamic non-CEA genes (*p* < 0.05), corresponding to 0.25% and 0.19% of all non-CEA gene/CpG pairs in each participant and each cell type, respectively. The same results were obtained in PBMCs; 242 (0.22%, participant #1) and 234 (0.27%, participant #2) CpG sites were associated with dynamic non-CEA genes (*p* < 0.05).

We also calculated the standard deviation (SD) of DNA methylation levels for each CpG locus. Notably, the median of the SDs of CpG methylation at loci located near dynamically expressed non-CEA genes was 0.01 in both participants’ monocytes (participant #1: IQR = 0.006–0.016, participant #2: IQR = 0.006–0.014); for PBMCs, the values was 0.008 (IQR = 0.005–0014) for participant #1 and 0.009 (IQR = 0.005–0.016) for participant #2. As observed with the distributions of the R^2^ value for linear regression and *p* values for the permutation test, the SD distribution for the pairs of dynamically expressed genes and neighboring CpG sites was not significantly different from that observed with non-dynamically expressed genes. These results showed that the intraindividual dynamics of DNA methylation was limited and unlikely to be associated with gene expression induced by external stimuli.

## Discussion

In the present study, we investigated to what extent DNA methylation contributes to dynamic changes in gene expression by collecting blood samples 24 times from two individuals over a 3-month period. Our analyses revealed that only ~2% of gene expression variation could be explained by intraindividual changes in DNA methylation in PBMCs and monocytes. Moreover, the number of associations detected between dynamic gene expression and DNA methylation was far fewer than the proportion of gene expression variance that can be explained by DNA methylation.

We stratified genes based on their association with the cell-type composition (Fig. 3, Table 1), which clearly showed that variations in cellular composition within a highly heterogeneous cell population—such as PBMCs—have a profound effect on the detection of actual changes in gene expression, irrespective of intracellular alterations (Table 2). Cell sorting was performed to mitigate this effect and accurately detect the expression dynamics representative of cell function. Accordingly, an increasing number of studies for epigenome characterization have been performed using single cell populations^10^,^11^,^12^,^13^.

Interestingly, the participants exhibited an elevated hs-CRP level at the time of the third and fifth blood collections, respectively, suggestive of moderate inflammation (>10 mg/L) (Fig. 1d). In the dynamic non-CEA genes enriched in monocyte function pathways (Table 2), this peak correlated with the increased expression of some genes involved in monocyte recruitment^14^ (CCL1, CCL8, CXCL10, CCR1) and antiviral activity (DDX58^15^, DHX58^16^, IRF7^17^, ISG15^18^, STAT1^19^, TNFSF10^20^, JAK3^21^) (Supplementary Fig. 1), indicating that antiviral responses were likely present at the time of the blood collections in each participant. Conversely, although the CRP levels were similar in both participants, there was a marked difference in the number of dynamic non-CEA genes correlated with the hs-CRP peak between the participants. This fact indicates that the expression of genes closely related to cell functions is extremely transient. These results suggested that DNA methylation hardly contributed to the variations in the dynamic expression necessary for such transient biological responses. Thus, intraindividual genome-wide DNA methylation can be considered to be quite stable for at least 3 months, even in the presence of acute biological responses. These results support the feasibility of epigenome-wide association studies, where genomic DNA methylation is often measured only once for each individual and the power to identify disease-associated CpG loci depends on the stability of the modification. Moreover, the stability of DNA methylation suggested that disease-associated DNA methylation markers would be useful for estimating the risk of disease manifestation.

## Methods

### Ethics statement

This study was approved by the Ethics Committee of Iwate Medical University (Approval ID: HG H25-1). All experiments were carried out in accordance with the approved guidelines. The study design was explained to both subjects, following which they provided written informed consent to participate in this study and provide anonymous samples.

### Blood collection and cell sorting

Both participants were healthy male volunteers in their thirties; they did not consume alcohol within 24 h prior to sample collection. Peripheral blood was collected from the participants after overnight fasting; the samples were collected in BD Vacutainer CPT tubes containing sodium heparin (8 mL; Becton Dickinson and Company, Franklin Lakes, NJ, USA) and a Venoject II AutoSep (gel + clot activator, 9 mL; Terumo, Tokyo, Japan) with a 21-gauge needle. The participants tracked their basal body temperature before blood collection. After the collection period, specimens were anonymized. Blood for serological tests was left to clot at room temperature for 30 min, centrifuged at 400 *g* for 20 min, and then frozen at −80 °C until analyses were performed by BML Inc. (Tokyo, Japan). PBMCs were separated by centrifugation (Sorvall Legend XFR; Thermo Fisher Scientific, Waltham, MA, USA) at 1,700 *g* for 20 min at room temperature. PBMCs were washed in 30 mL phosphate buffer saline containing 2 mM EDTA, and then centrifuged at 250 *g* for 10 min at room temperature to remove any contaminating platelets and plasma. Isolated PBMCs were then counted using a C-Chip disposable hemocytometer (Biochrom AG, Berlin, Germany) and resuspended at 5 × 10^6^ cells/mL.

PBMCs were stained with 10 μL CD14-FITC and CD16-PE antibody (Sony Biotechnology, Inc., Tokyo, Japan) and incubated for 20 min at 4 °C. After washing with a 5× volume of PBS, the sample was immediately analyzed using a CellSorter SH800 (Sony). CD14^high^CD16^low^ monocytes were sorted from the monocyte-containing gate determined by light-scatter density-plot, and then analyzed for CD14 and CD16 expression (Fig. 1b).

Genomic DNA and RNA were extracted from the 24 PBMC and monocyte samples using the AllPrep DNA/RNA Micro Kit (QIAGEN, Venlo, Netherlands) according to the manufacturer’s instructions. Genomic DNA and RNA yields were measured using the Qubit 2.0 Fluorometer (Life Technologies, Carlsbad, CA, USA) with the Qubit dsDNA BR and Qubit RNA Assay Kit, respectively. Genomic DNA integrity was assessed using Genomic DNA ScreenTape on an Agilent 2200 TapeStation (Agilent Technologies, Santa Clara, CA, USA).

### Preparation of total RNA library and RNA sequencing

We prepared cDNA libraries from 150 ng of total RNA with TruSeq RNA sample Preparation Kit v2 (Illumina, San Diego, CA, USA) for the RNA-Seq, and then carried out fragment size and expression analyses as previously described^22^. For RNA-Seq, equimolar mixtures of the twelve libraries were loaded into four flow cells for cluster generation using a TruSeq Rapid SR Cluster Kit-HS (Illumina) and sequenced on a HiSeq2500 system (Illumina) using a TruSeq Rapid SBS Kit-HS (Illumina), generating 1 × 101 bp reads. Sequencing was performed on the samples from the same individuals in the same batch.

### DNA methylation profiling using Illumina bead arrays

Genomic DNA samples (500 ng each) were bisulfite-converted with an EZ DNA methylation kit (Zymo Research Corporation, Irvine, CA, USA), amplified, and then hybridized to the array chips, followed by staining and processing of the single-base extension using HM450 BeadChip Kit (Illumina) according to the manufacturer’s instructions. Chips were imaged by iScan (Illumina). Methylation profiling was performed on the samples from the same individuals in the same batch.

### Bioinformatics and statistical analysis

#### RNA-Seq data analysis

Quality assessment and quality control of reads were evaluated using FastQC^23^ (Babraham Informatics, Cambridgeshire, UK). Reads were annotated according to the human genome index with Bowtie-build v0.12.9^24^ and mapped to the human reference genome (hs37d5) using TopHat v.2.0.9^25^ and SAMtools v0.1.19^26^. The expression values of reads with upper-quartile normalization of the fragments per kilobase of transcript per million fragments mapped reads (FPKM) were calculated in Cufflinks/Cuffdiff v2.1.1^27^. The data normality was evaluated by the Shapiro-Wilk test.

#### DNA methylation data analysis

Raw DNA methylation data were processed using the R environment (v.3.0.2) and the Bioconductor lumi package (version 2.14.2)^28^. In this study, we quantified methylation based on beta values ranging from 0 to 1, defined as the ratio of methylated probe intensity to total signal intensity^29^. After loading of raw intensity data (IDAT) files, color balance adjustment, background level correction, and quantile normalization were performed for signal intensities as implemented in lumi. Then, probe type bias adjustment was performed with normalized data using beta-mixture quantile normalization on beta values^30^. The quality of methylation assays was assessed by mean detection *P*-value. Probes whose detection *p-*values were <0.01 were retained for further analysis. To estimate cell-type composition, the “estimateCellCounts” function^31^ implemented in the minfi package was modified to use values of the lumi- and beta-mixture quantile-normalized beta value table as the input^32^. The data normality was evaluated by the Shapiro-Wilk test.

#### Detection of dynamically expressed genes

The average of log_10_ [FPKM + 1] of each gene was separated into bins with 0.05 intervals. For each bin, coefficient of variation outliers for each gene were detected by Smirnov-Grubbs test and defined as dynamically expressed genes.

#### *Cell-type composition/Expression* a *ssociation* (*CEA*) *analysis*

To exclude the genes sensitive to cell-type composition, we performed an association test between gene expression levels and cell-type composition. Cell-type composition was estimated from the methylation profile for each time point (see also “DNA methylation data”). Then, for each gene, a linear regression model, e.g., 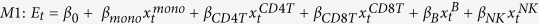, was applied to our 24 transcriptome data points, where E_t_ represents the expression level (log_10_ [FPKM+1]) of the gene at a time point *t*, 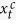 represents the proportion of the cell type *c* at the time point *t*, *β*_0_ represents the intercept, and *β*_*c*_ represents the coefficient for the cell type *c*. As the null model, we assumed a model 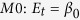. Then, two models M0 and M1 were compared and the *p*-value was calculated by ANOVA. The lower *p*-values for the cell-type composition variables explain increasing variance in gene expression.

#### Pathway enrichment analysis

Pathway enrichment analysis was performed on DAVID Bioinformatics Resources (version 6.7) using the extract gene list. Enriched pathways were defined by a Bonferroni *p-*value less than 10^−4^ and a false discovery rate less than 0.01^33^.

#### *Methylation/e* xpression a *ssociation* (*MEA*) *analysis*

To investigate the extent to which DNA methylation contributes to the dynamics of gene expression, we first extracted pairs of genes and neighboring CpG sites (within 5,000 bases from the transcription start site), according to the RefGene annotation (Illumina manifest v1.1), resulting in 273,660 gene-CpG pairs. We excluded the CpG sites that are consistent with the SNP sites by using Infinium HD Methylation SNP List (Illumina, humanmethylation450_dbsnp137.snpupdate.table.v2.sorted.txt).

For each pair, we performed linear regression analysis with or without adjustment of cell-type composition. Model 1: 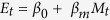, and Model 2: 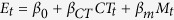, where E_t_ represents the expression level (ratio of log_10_ [FPKM + 1] to that at Day 1) of the gene at a time point *t*, M_t_ represents the methylation level (ratio of beta value to that at Day 1) of the CpG site at a time point *t*, CT_t_ represents the variation of population of the cells that provided the lowest p value by CEA, *β*_0_ represents the intercept, *β*_*m*_ represents the coefficient of the methylation level, and *β*_*CT*_ represents the coefficient of variation of the cell population. Then, the R^2^ value of the model represents the proportion of the gene expression variance that can be explained by DNA methylation. The R^2^ value of Model 2 was calculated by the squared correlation coefficient between 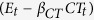 and *β*_*m*_*M*_*t*_.

We also obtained *p* values for the Spearman’s rank correlations of the expression level with the methylation level on the basis of a permutation test; we performed 1,000 random permutations of the expression level of the gene at a time point.

## Additional Information

**How to cite this article**: Furukawa, R. *et al.* Intraindividual dynamics of transcriptome and genome-wide stability of DNA methylation. *Sci. Rep.* **6**, 26424; doi: 10.1038/srep26424 (2016).

## Supplementary Material

Supplementary Information

## Figures and Tables

**Table 1:** Summary of cell-type composition/expression associations.

	CEA		non-CEA	
	dynamic	non-dynamic	dynamic	non-dynamic
(a) Monocytes				
Participant #1	69	3752	379	15386
Participant #2	150	3138	361	15937
(b) PBMCs				
Participant #1	149	6688	180	13569
Participant #2	225	5707	225	13429

**Table 2:** Pathway enrichment analysis of dynamic non-CEA genes.

Participant	Term	Gene	*P*-value	Fold enrichment	Bonferroni	FDR
(a) Monocytes						
Participant #1	hsa04060:Cytokine-cytokine receptor interaction	CXCL1, CCL3, TNF, CCL2, CXCL3, CCR1, CXCL2, CCL8, PF4, CCL4, IL10, CXCL10, CCL24, CXCR4, CCL3L3, IL1B, IL8, FLT3, CCL4L2, OSM, TNFSF10, PPBP, CX3CR1, CCR2, VEGFA	2.72E-20	8.07	2.67E-18	3.01E-17
	hsa04062:Chemokine signaling pathway	CXCL1, CCL3, CCL2, IL8, CCR1, CXCL3, CXCL2, NFKBIA, CCL8, PF4, CCL4L2, STAT1, CCL4, CXCL10, STAT2, CCL24, PPBP, CXCR4, CX3CR1, CCL3L3, CCR2, RAP1A	8.38E-19	8.58	8.22E-17	9.27E-16
	hsa04010:MAPK signaling pathway	TNF, DUSP10, NR4A1, HSPA1A, HSPA1B, DDIT3, JMJD7-PLA2G4B, DUSP2, JUN, JUND, HSPA6, MAPK8IP3, IL1B, RAP1A, GADD45B, MYC, DUSP7	1.42E-14	9.36	1.39E-12	1.57E-11
	hsa04620:Toll-like receptor signaling pathway	CCL3, TNF, IL8, JUN, IRF7, NFKBIA, IL1B, FADD, STAT1, CCL4, CXCL10	6.69E-09	8.58	6.55E-07	7.39E-06
	hsa04621:NOD-like receptor signaling pathway	CXCL1, TNF, CCL2, IL8, CXCL2, CCL8, NFKBIA, IL1B, RIPK2, TNFAIP3	5.68E-08	8.51	5.56E-06	6.28E-05
	hsa04622:RIG-I-like receptor signaling pathway	DDX58, TNF, ISG15, IL8, IRF7, NFKBIA, FADD, DHX58, CXCL10	1.03E-07	9.36	1.01E-05	1.14E-04
	hsa04610:Complement and coagulation cascades	C1QA, C3AR1, C5AR1, F3, SERPING1, C1QC, PLAUR	7.97E-06	9.36	7.80E-04	0.0088
Participant #2	hsa04062:Chemokine signaling pathway	CXCL1, CCL3, DNAJC25-GNG10, CCL2, IL8, CXCL2, NFKBIA, CCL4, CCNL2, STAT2, GNG8, CCL20, CXCR4, RAP1A, PIK3R5, JAK3, PLCB2	2.02E-08	5.71	2.18E-06	2.27E-05
	hsa04621:NOD-like receptor signaling pathway	CXCL1, CCL2, IL8, CXCL2, NFKBIA, IL1B, TNFAIP3, NLRP1	4.49E-05	8.10	4.83E-03	5.05E-02
	hsa04060:Cytokine-cytokine receptor interaction	CXCL1, ZFP91-CNTF, CCL3, CCL2, IL8, FLT3, CXCL2, CCL4, IL11RA, CCNL2, OSM, CCL20, CXCR4, IL1B	1.89E-04	3.35	2.02E-02	2.13E-01
(b) PBMCs						
Participant #1	hsa04060:Cytokine-cytokine receptor interaction	OSM, TNFRSF6B, CCL3, CNTF, IL8, CXCR4, TNFRSF25, CXCL2, CCL3L3, TNFSF12-TNFSF13	5.98E-07	7.50	3.83E-05	6.08E-04

There was no enriched pathway in the dynamic non-CEA genes in PBMCs from participant #2.
