# Supplementary material for: Intraindividual dynamics of transcriptome and genome-wide stability of DNA methylation
Source: Sci Rep. 2016 May 19;6:26424. doi: 10.1038/srep26424 (PMC4872231; doi:10.1038/srep26424)
Supplement: Supplementary Information [file srep26424-s1.pdf]

Intraindividual dynamics of transcriptome and genome-wide stability of DNA methylation

Ryohei Furukawa, Tsuyoshi Hachiya, Hideki Ohmomo, Yuh Shiwa, Kanako Ono, Sadafumi Suzuki, Mamoru Satoh, Jiro Hitomi, Kenji Sobue, Atsushi Shimizu

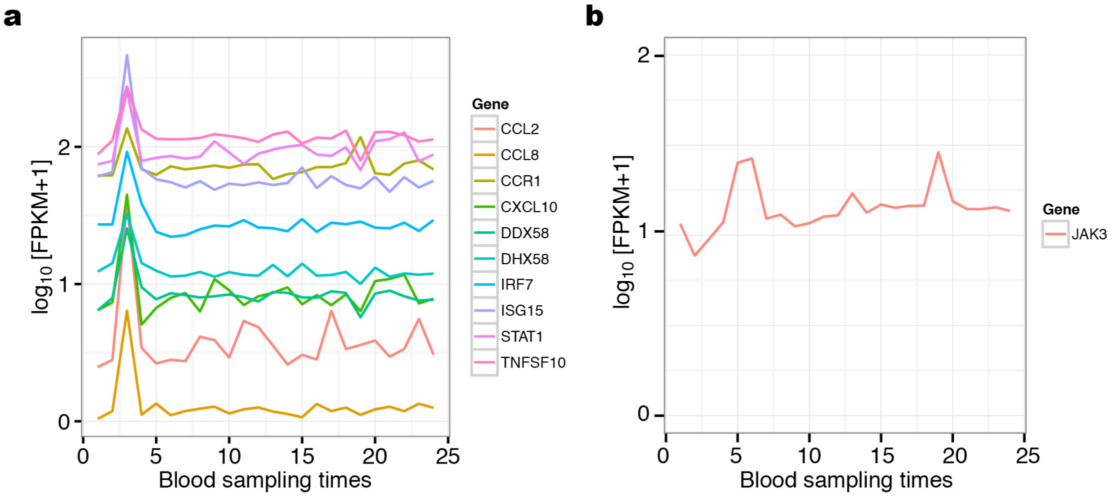

Supplementary Figure 1: Variation in the expression of the dynamic non-CEA genes, coincident with the change in C-reactive protein levels. The increased expression peak for a subset of genes enriched in the innate immune pathways was coincident with a peak in serum CRP (See Fig. 1d). (a) Participant #1. (b) Participant #2.

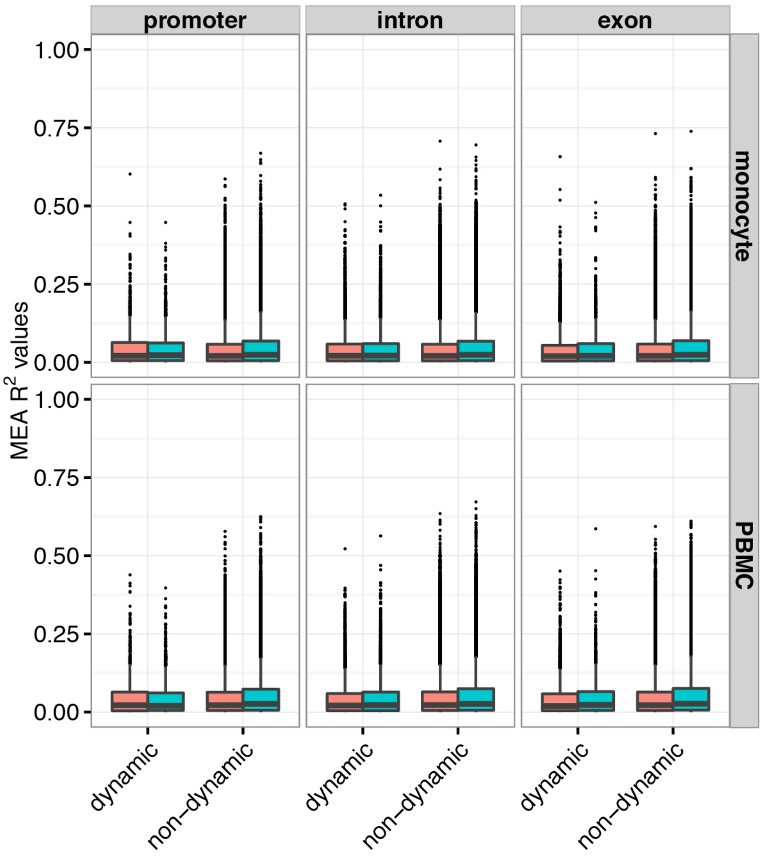

Supplementary Figure 2: Contribution of DNA methylation to non-CEA gene expression dynamics in each genomic region. The distribution of the R^2 values was computed by linear regression analysis with the model $E_i = \beta_{0i} + \beta_{1i} M_i$. The gene expression and DNA methylation levels were converted to relative values using the values of Day 1 as a reference.

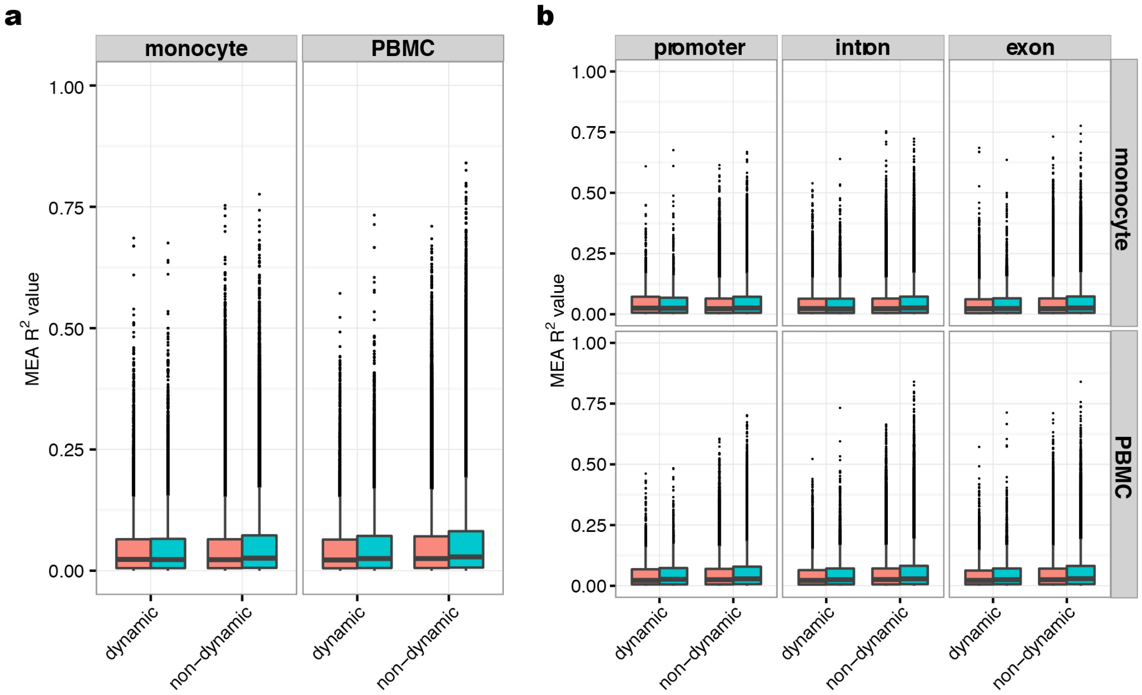

Supplementary Figure 3: Contribution of DNA methylation to gene expression dynamics. The R^2 values were calculated by linear regression analysis with adjustment of cell composition ($E_i = \beta_0 + \beta_{ct} CT_i + \beta_m M_i$). (a) The distribution of the R^2 values of all CpG/gene pairs. (b) The distribution of the R^2 values of the CpG/gene pairs in each genomic region.

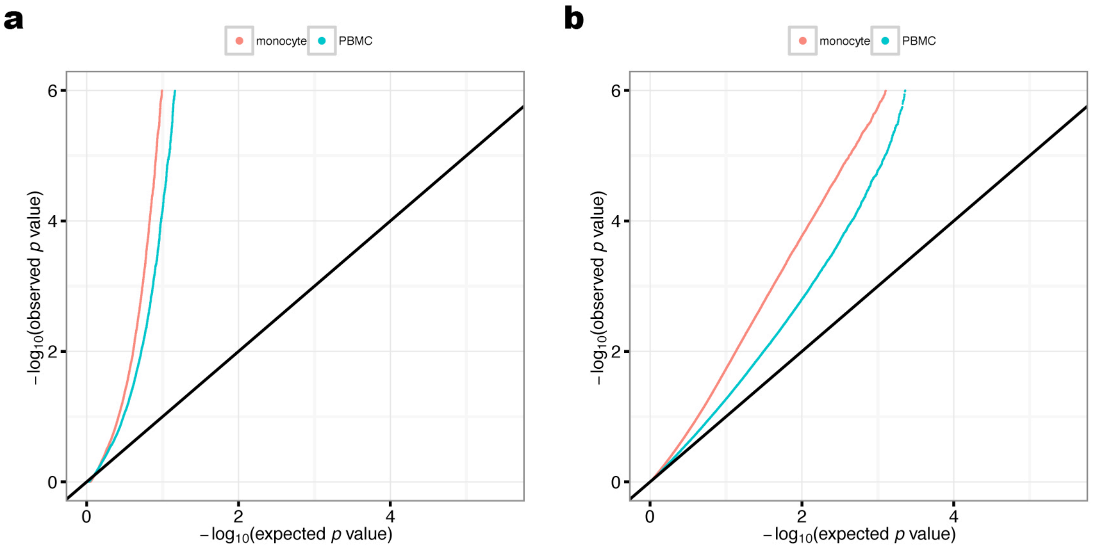

Supplementary Figure 4: Evaluation of the normality of the gene expression and DNA methylation data. Quantile-quantile plot for the comparison of gene expression (a) and DNA methylation (b) between the time points. The *p* values were obtained from the Shapiro-Wilk test.

Supplementary Table 1: Serological analysis results for the participants

Analyte	*Participant 1 (mean ± SD)*	*Participant 2 (mean ± SD)*
GOT (U/L)	16.7 ± 3.0	19.1 ± 1.3
GPT (U/L)	13.5 ± 3.5	18.3 ± 2.2
γ-GTP (U/L)	15.1 ± 1.3	13.5 ±1.9
creatinine (mg/dL)	0.7 ± 0.0	0.8 ± 0.0
uric acid (mg/dL)	6.3 ± 0.5	4.1 ± 0.3
uric acid nitrogen (mg/dL)	9.9 ± 1.0	11.2 ± 8.2
total cholesterol (mg/dL)	189.5 ± 9.0	163.3 ± 9.2
HDL cholesterol (mg/dL)	47.6 ± 2.8	40.9 ± 2.3
LDL cholesterol (mg/dL)	119.9 ± 10.1	98.7 ± 8.2
triglyceride (mg/dL)	159.3 ± 36.6	136.5 ± 25.3
total IgE antibody (IU/mL)	365.6 ± 33.5	81.1 ± 9.2

Supplementary Table 2: Summary of cell-type composition/expression associations with the excluded data for the day with high HSCRP level

(a) Monocytes

	CEA		non-CEA	
	dynamic	non-dynamic	dynamic	non-dynamic
Participant #1 (without Day 3)	50	1474	380	17682
Participant #2 (without Day 5)	78	3267	106	16135

(b) PBMCs

	CEA		non-CEA	
	dynamic	non-dynamic	dynamic	non-dynamic
Participant #1 (without Day 3)	64	2778	208	16536
Participant #2 (without Day 5)	241	5606	54	13685

Supplementary Table 3: Pathway enrichment analysis of dynamic non-CEA genes

(a) Monocytes

Participant	Term	Genes	*P*-value	Fold Enrichment	Bonferroni	FDR
Participant #1 (without Day 3)	hsa04060:Cytokine-cytokine receptor interaction	CXCL1, CCL3, TNF, IL8, FLT3, CXCL3, CXCL2, PF4, CCL4L2, CCL4, CXCL10, CCL24, OSM, TNFRSF1A, PPBP, CXCR4, CXCL16, VEGFA, CX3CR1, CCL3L3, CCR2, IL1B	1.43E-07	3.81	1.54E-05	1.61E-04
	hsa04010:MAPK signaling pathway	TNF, DUSP10, NR4A1, HSPA1A, HSPA1B, DDIT3, DUSP5, TNFRSF1A, FOS, ATF4, JMJD7-PLA2G4B, DUSP2, DUSP1, JUN, MAP3K8, JUND, HSPA6, RAP1A, HSPA7, IL1B, GADD45B, MYC, DUSP7, DUSP6	1.98E-07	3.74	2.13E-05	2.23E-04
	hsa04062:Chemokine signaling pathway	CXCL1, CCL3, IL8, CXCL3, CXCL2, NFKBIA, PF4, CCL4L2, CCL4, CXCL10, CCL24, PPBP, CXCR4, CXCL16, CX3CR1, CCL3L3, CCR2, RAP1A	4.37E-07	4.37	4.72E-05	4.92E-04
	hsa04620:Toll-like receptor signaling pathway	FOS, CCL3, TNF, IL8, JUN, MAP3K8, NFKBIA, IL1B, FADD, CCL4, CXCL10	5.95E-05	4.94	6.41E-03	6.70E-02
	hsa04621:NOD-like receptor signaling pathway	CXCL1, TNF, IL8, CXCL2, NFKBIA, IL1B, RIPK2, TNFAIP3	3.57E-04	5.86	3.78E-02	4.01E-01
Participant #2 (without Day 5)	hsa04062:Chemokine signaling pathway	GNG8, CCL24, DNAJC25-GNG10, CCL2, CCL20, CXCL3, CCL8	1.04E-04	8.28	4.26E-03	9.58E-02
	hsa04060:Cytokine-cytokine receptor interaction	ZFP91-CNTF, CCL24, TNF, CCL2, CCL20, CXCL3, CCL8	6.55E-04	5.91	2.65E-02	6.01E-01

(b) PBMCs

Participant	Term	Genes	*P*-value	Fold Enrichment	Bonferroni	FDR
Participant #1 (without Day 3)	hsa04062:Chemokine signaling pathway	GNG8, CCL3, CCL2, PPBP, IL8, CXCR4, CCL3L3, NFKBIA, GNG11, PF4	3.44E-05	5.79	2.16E-03	3.48E-02
	hsa04060:Cytokine-cytokine receptor interaction	OSM, CCL3, CCL2, PPBP, IL8, CXCR4, TNFRSF25, CCL3L3, TNFRSF17, PF4, TNFSF12-TNFSF13	8.70E-05	4.54	5.47E-03	8.81E-02

There was no enriched pathway in the dynamic non-CEA genes in PBMCs from participant #2.
